# Evaluation of the Jokoo-ION 150 AC: Guidelines for the evaluation of analysers by ion-selective electrodes

**DOI:** 10.1155/S1463924690000153

**Published:** 1990

**Authors:** Joan Farré, Carmen Biosca, Román Galimany

**Affiliations:** Servei d'Anàlisis Clíniques Hospital de Badalona ‘Germans Trias i Pujol’ Apartado de Correos, 72 Can Ruti., Badalona Barcelona 08916 Spain

## Abstract

The Jokoo-ION 150 AC is an automatic sodium, potassium and chloride analyser which uses ion-selective electrodes.

The sample mode can be whole-blood, serum or urine. To evaluate a urine sample, a previous dilution (1:6) with the standard 1 solution is required. For three concentrations of control materials, the total precision (CV) ranged from 0.17 to 1.22% for sodium, 0.22 to 1.69% for potassium and 0.16 to 0.74% for chloride.

The system demonstrated acceptable performance in detection limit, linearity, drift and carry-over. Patients' results from ION 150 AC correlated well with those from a SMAC II and an IL 943. In a study on potential interferences, a slightly negative interference with potassium was found with increases in lithium; and only high ammonium ion concentrations caused a positive interference on the potassium ion. A slightly positive interference of bromide on the chloride ion was also found.

The electrode slope, electrode response, sample temperature and pH effect, effects of high concentrations of proteins or lipids, and haematocrit influence on the sodium, potassium and chloride ion concentration were also evaluated. The strategy adopted in this study provides an ideal framework for future evaluations of ionselective electrode analysers.


Journal of Automatic Chemistry, Vol. 12, No. 3 (May–June 1990), pp. 116–128

### Evaluation of the Jokoo-ION 150 AC: Guidelines for the evaluation of analysers by ion-selective electrodes

**Joan Farré, Carmen Biosca and Román Galimany**

*Servei d'Anàlisis Clíniques, Hospital de Badalona 'Germans Trias i Pujol', Apartado de Correos, 72, Can Ruti., 08916 Badalona, Barcelona, Spain*

*The Jokoo-ION 150 AC is an automatic sodium, potassium and chloride analyser which uses ion-selective electrodes.*

*The sample mode can be whole-blood, serum or urine. To evaluate a urine sample, a previous dilution (1:6) with the standard 1 solution is required. For three concentrations of control materials, the total precision (CV) ranged from 0·17 to 1·22% for sodium, 0·22 to 1·69% for potassium and 0·16 to 0·74% for chloride.*

*The system demonstrated acceptable performance in detection limit, linearity, drift and carry-over. Patients' results from ION 150 AC correlated well with those from a SMAC II and an IL 943. In a study on potential interferences, a slightly negative interference with potassium was found with increases in lithium; and only high ammonium ion concentrations caused a positive interference on the potassium ion. A slightly positive interference of bromide on the chloride ion was also found.*

The electrode slope, electrode response, sample temperature and pH effect, effects of high concentrations of proteins or lipids, and haematocrit influence on the sodium, potassium and chloride ion concentration were also evaluated. The strategy adopted in this study provides an ideal framework for future evaluations of ion-selective electrode analysers.

### Introduction

The device which is commonly called an 'ion-selective electrode' ('ISE') determines the activity (A) of an ion in solution (in clinical biochemistry it usually refers to cations). The essential part of this device is a membrane with properties related to the ion being evaluated: it is specifically, or at least selectively, permeable to this ion. Therefore, a potential difference is established between both sides of the membrane, which is proportional to the ion activity difference. The technology behind electrometric instruments aims to make the difference in potential as small as possible.

As a result, a measuring chain is used where a series of different devices are capable of exploiting this small potential. These complementary devices are called reference electrodes, and are characterized by the stability of their own potential. There are two classes of reference electrode; silver chloride and mercury chloride (calomel). The chain of measurement is composed of a sequence including a reference electrode (called 'external') and a measuring electrode – the latter being a reference electrode designated 'internal', which is separated from the selective membrane by an electrolytically simple solution of the ion to be evaluated. The difference of potential measured at the extreme points of the system is derived from the equation of Nernst:

$$E = E_0 + E_p + 2{\cdot}3\,\frac{RT}{nF}\log A \qquad [1]$$

$E_0$ is the sum of the fixed potentials depending on the measuring and reference electrodes structure, $E_p$ is the sum of the variable potentials, 'parasites', that have to be annulled, minimized or at least stabilized, $R$ is the perfect gases constant, $T$ is absolute temperature, $F$ is Faraday's number, and $n$ is valence.

$$\begin{aligned} A &= \gamma \times C \\ \log\gamma &= 0{\cdot}509\, n^2 \sqrt{I} \\ I &= 1/2\, \Sigma(C_i n_i^2) \end{aligned} \qquad [2]$$

where $C_i$ is concentration, $n_i$ is valence (of each ion present in the solution), $A$ is the activity, $\gamma$ is the activity coefficient, $C$ is the molar concentration, and $I$ is the ionic force.

At very dilute concentrations ($10^{-7}$ M), the activity coefficient is very near to unity but at higher concentrations it diminishes [3 and 14]. Thus, the calibrator must have an activity coefficient similar to that of the sample.

Ion-selective electrodes have a big international market due to their simplicity in use and their inclusion in automatic analysers.

The unequal quality of the sensors currently available, together with the sparse experience that analysis laboratories have in the field of electrometry and the limited quality control materials at present available from manufacturers, means that these instruments are often difficult to work with.

The present paper presents an evaluation of an ion-selective electrode analyser (the Jokoo-ION 150 AC) and suggests guidelines for studying, controlling and evaluating ion-selective electrode analysers.

### Materials and methods

*Automatic analyser SMAC II (Technicon Instruments Corporation, Tarrytown, New York 10591, USA)*

SMAC II is a continuous flow analyser, which evaluates sodium and potassium ions by indirect potentiometry. For sodium the total dilution is 1:16·2, and for potassium 1:18·8.

0142–0453/90 $3.00 © 1990 Taylor & Francis Ltd.

The sodium ion utilizes a sodium–aluminium glass electrode where sodium selectivity against potassium amounts to 1000:1; the serum sample is buffered at pH 8·0 to eliminate any interference by the hydrogen ion [4].

The Valynomicine electrode used for the potassium ion has a selectivity of nearly 4000:1 against the sodium ion. Valynomicine is an antibiotic which is insoluble in water, but is soluble in organic solvents, such as ether or acetone. It is suspended at the end of the electrode in a porous membrane. The inner solution, formed by potassium chloride, is in contact with the Ag–ClAg inner medium from the reference electrode [5].

The evaluation of the chloride ion uses a mercuric thiocyanate colorimetric method based on Zall *et al.*'s manual method, automated by Skeggs and later modified by Morgenstern *et al.* The absorbance reaction is measured at 480 nm [6].

*IL 943 flame photometer (Instrumentation Laboratory, Paderno, Dugnano, Italy)*

The IL 943 has a dilutor incorporated, providing automatic calibration and sampling. The calibrator and sample dilution is 1:100. It is made with a caesium chloride solution, which has final concentration of 1·5 mmol/l. The instrument's zero is regulated with distilled water at 1:100 with the inner standard (caesium chloride solution [7]. The gas used is 99% pure propane at 3·6 psi.

*Jokoo-ION 150 AC analyser*

This analyser measures sodium, potassium and chloride ion concentrations simultaneously for every sample through selective electrodes by direct potentiometry.

The sample volume needed is 100 μl, plus 50 μl residual volume. This sample can be whole-blood, plasma or urine. To evaluate a urine sample, a previous dilution (1:6) with standard 1 solution is required. The throughput is 150 samples/hour.

When the system is ready, a complete calibration is realized, being recalibrated automatically at two points every 30 min (this interval is optional).

The automatic sampler holds 30 sample cuvettes in a gyratory disk, allowing positional identification of the sample. This unit can use manual sampling through syringes or capillaries, and stat sampling. It has an automatic sample detector. Results are obtained in visual digits and are also printed-out.

The sample is aspirated through a capillary and is then transported directly to the electrode unit by a peristaltic pump which is placed after the measuring unit. The capillary is rinsed automatically with standard solution 1.

There are four electrodes in the measuring unit: sodium, potassium, chloride and the reference electrode. The reference electrode system is a double union type (liquid–liquid) (Ag/AgCl in saturated KCl solution), which provides a stable potential that can be used as a reference. The sodium electrode is a glass membrane electrode (sodium aluminium silicate). The potassium electrode is a liquid membrane electrode, which is based on a neutral carrier (Valynomicine-PVC, polyvinyl chloride as a support). Finally, the chloride electrode is a liquid membrane electrode, which is based on ionic exchange using quaternary ammonium salts as an ion-exchanger in polymeric solvent.

Also used in the evaluation were a tester (Kaiser model SK-6300); a voltimeter of $10^{14}$ Ohms and a sensitivity of 0·1 milivolt (mV); and an analogous registrator (Linear Instruments Corporation Model 1201; scale 1 mV–5 V).

*Calibrating solutions*

For the SMAC II calibration two seric multiple constituent calibrators at two different concentrations were used: 144 mmol/l sodium, 6·1 mmol/l potassium and 112 mmol/l chloride (calibrator 1); 100 mmol/l sodium, 3 mmol/l potassium and 85 mmol/l chloride (calibrator 2) (Technicon Instruments Corporation).

For the flame photometer calibrations a water calibrator was used, which was common to both ions, sodium and potassium, in concentrations of 140 mmol/l sodium and 5 mmol/l potassium, for serum samples (Il catalogue number 97.517-50). For urine samples, the calibrator concentration runs up to 100 mmol/l of sodium and 100 mmol/l of potassium (Il catalogue number 35.100); whilst, for the lithium ion calibration, the concentration runs up to 140 mmol/l sodium, 5 mmol/l potassium and 1 mmol/l lithium (Il catalogue number 33.313-50). To calibrate the Jokoo-ION 150 AC analyser, two solutions were used: $C_1$ = lot 0625397, with 140 mmol/l sodium, 4 mmol/l potassium, 100 mmol/l chloride; and, $C_2$ = lot 0918407, with 160 mmol/l sodium, 6 mmol/l potassium and 120 mmol/l chloride.

*Controls*

Three commercial lyophilized controls of human origin at three concentrations (low, medium and high) were used. These were selected to represent common clinical decisions. The manufacturer's recommendations were followed to reconstitute the lyophilized control. A pool was prepared and stored in aliquot parts in the freezer (−20 °C). As urine control, a pool of urine was used to study the within-run imprecision.

*Reagents*

(1) Kit for determination of ammonium in blood (SIGMA Diagnostics, St. Louis, USA). This method is based on the ammination of 2-oxoglutarate by means of glutamate dehydrogenase (GLDH) and reduced nicotinamide adenine dinucleotide (NADH). The absorbance decrease, measured at 340 nm, as a result of the NADH oxidation, is proportional to the concentration of plasma.

(2) Buffer solution Tris-hydroxymethyl aminomethane orthophosphoric acid pH 7·35 (Tris 0·2 M, orthophosphoric acid 0·15 N).

(3) Human albumin Hubber at 20% (ref. 702811).
(4) Lipaemic solution at 10% Tutolipid (ref. 963769).
(5) Lithium heparin salt (vacutainer tubes).
(6) Sodium chloride (Merck art. 6404, 99·5% purity)
(7) Potassium chloride (Merck art. 4937, 99·5% purity).
(8) Ammonium chloride (Merck art. 1145, 99·8% purity).
(9) Brij-35, 30% (Technicon product No. T-21-0110).
(10) Bidistilled and deionized water.
(11) Potassium bromide (Merck art. 4905, 99·5% purity).
(12) TRIS (Merck art. 8382, 99·5% purity).
(13) Orthophosphoric acid (Merck art. 565, 98% purity).

### Results

### *Imprecision*

*Within-run imprecision*

A sample of each of the three controls was analysed 20 times in the same run without interruption for calibration or recovery of the base-line. This process was repeated for two working days. The mean, standard deviation (SD) and the coefficient of variation (CV) on each concentration level were calculated.

The urine pool was prepared in the laboratory; it was diluted at 1:6 in standard solution 1, and was evaluated 30 times within the same run/test. The mean, SD and CV were also calculated.

The results are shown in table 1: precision for the three ions for all three concentration levels was high as was precision with the urine pool.

*Table 1. Within-run imprecision.*

| Serum samples | $N = 100$ | First series | Second series | Third series |
|---|---|---|---|---|
| Sodium | | | | |
| Low level | $\bar{x}$ (mmol/l) | 118 | 120 | 120 |
| | CV (%) | 0·29 | 0·47 | 0·35 |
| Medium level | $\bar{x}$ (mmol/l) | 140 | 138 | 140 |
| | CV (%) | 0·2 | 0·19 | 0·21 |
| High level | $\bar{x}$ (mmol/l) | 149 | 154 | 155 |
| | CV (%) | 0·37 | 0·27 | 0·17 |
| Potassium | | | | |
| Low level | $\bar{x}$ (mmol/l) | 3·1 | 3·1 | 3·1 |
| | CV (%) | 0·23 | 0·63 | 0·47 |
| Medium level | $\bar{x}$ (mmol/l) | 6·5 | 6·4 | 64 |
| | CV (%) | 0·39 | 0·22 | 0·2 |
| High level | $\bar{x}$ (mmol/l) | 7·2 | 7·3 | 7·3 |
| | CV (%) | 0·37 | 0·22 | 0·33 |
| Chloride | | | | |
| Low level | $\bar{x}$ (mmol/l) | 96 | 95 | 95 |
| | CV (%) | 0·25 | 0·17 | 0·16 |
| Medium level | $\bar{x}$ (mmol/l) | 119 | 116 | 116 |
| | CV (%) | 0·29 | 0·23 | 0·19 |
| High level | $\bar{x}$ (mmol/l) | 127 | 125 | 126 |
| | CV (%) | 0·47 | 0·6 | 0·74 |

| | Sodium | | Potassium | | Chloride | |
|---|---|---|---|---|---|---|
| Urine $N = 30$ | $\bar{x}$ (mmol/l) | CV (%) | $\bar{x}$ (mmol/l) | CV (%) | $\bar{x}$ (mmol/l) | CV (%) |
| | 65 | 2 | 26·1 | 0·47 | 77 | 1·85 |

*Day-to-day imprecision*

One sample of each of the three control concentration levels was evaluated each day over 20 days. The mean, SD and CV were computed for each of the three concentration levels. The results are presented in table 2. A high precision for the three components in the three studied concentration levels is shown.

*Table 2. Day-to-day imprecision study.*

| | Sodium | | Potassium | | Chloride | |
|---|---|---|---|---|---|---|
| Serum $N = 20$ | $\bar{x}$ (mmol/l) | CV (%) | $\bar{x}$ (mmol/l) | CV (%) | $\bar{x}$ (mmol/l) | CV (%) |
| Low | 121·39 | 1·22 | 3·12 | 0·69 | 96·21 | 0·62 |
| Medium | 139·14 | 0·84 | 6·31 | 1·02 | 116·88 | 0·47 |
| High | 153·45 | 0·64 | 7·19 | 1·69 | 125·14 | 0·53 |

### *Inaccuracy*

Because there were no commercial controls available for evaluating the system, primary standards at three concentration levels were prepared by weighing them into buffer Tris-orthophosphoric acid (Tris 0·2 M, orthophosphoric acid 0·15 N), adjusted to pH 7·35, and adding a surfactant (brij 30%) at 1 ml/l. Table 3 shows the concentration levels which were examined. A duplicate determination was carried out and the theoretical value was checked by flame photometry. To calculate the inaccuracy percentage, the following formula was used:

*Table 3. Inaccuracy study.*

| Primary standard $N = 20$ | Measured (mmol/l) | Theoretical (mmol/l) | Inaccuracy (%) |
|---|---|---|---|
| Sodium | | | |
| Low | 118·16 | 120 | −1·53 |
| Medium | 135·06 | 140 | −3·53 |
| High | 154·65 | 160 | −3·60 |
| Potassium | | | |
| Low | 2·41 | 2·5 | −3·6 |
| Medium | 3·84 | 4 | −4 |
| High | 5·82 | 6 | −3 |
| Chloride | | | |
| Low | 110·67 | 122·5 | −9·66 |
| Medium | 131·01 | 144 | −9·02 |
| High | 151·86 | 166 | −8·52 |

*Table 4. Relative inaccuracy.*

*(a) Correlation between indirect potentiometry and chloride determination (SMAC II) and direct potentiometry (Jokoo-ION 150 AC).*

| $N = 100$ | Correlation coefficient | Least square | Deming's regression |
|---|---|---|---|
| Sodium (serum) | 0·9604 | $a = 23{\cdot}514$ | $a = 17{\cdot}76$ |
| | | $b = 0{\cdot}815$ | $b = -0{\cdot}856$ |
| Potassium (serum) | 0·9914 | $a = 0{\cdot}028$ | $a = -0{\cdot}045$ |
| | | $b = 0{\cdot}971$ | $b = 0{\cdot}987$ |
| Chloride (serum) | 0·9643 | $a = 11{\cdot}121$ | $a = 4{\cdot}515$ |
| | | $b = 0{\cdot}902$ | $b = 0{\cdot}968$ |

*(b) Correlation between flame photometry (IL 943) and direct potentiometry (Jokoo-ION 150 AC).*

| $N = 100$·(serum) $N = 90$ (urine) | Correlation coefficient | Least square | Deming's regression |
|---|---|---|---|
| Sodium (serum) | 0·9752 | $a = 17{\cdot}568$ | $a = 13{\cdot}052$ |
| | | $b = 0{\cdot}866$ | $b = 0{\cdot}899$ |
| Potassium (serum) | 0·9827 | $a = -0{\cdot}15$ | $a = -0{\cdot}312$ |
| | | $b = 1{\cdot}04$ | $b = 1{\cdot}076$ |
| Sodium (urine) | 0·9789 | $a = 23{\cdot}994$ | $a = 22{\cdot}493$ |
| | | $b = 0{\cdot}801$ | $b = 0{\cdot}820$ |
| Potassium (urine) | 0·9933 | $a = 2{\cdot}222$ | $a = 2{\cdot}095$ |
| | | $b = 0{\cdot}829$ | $b = 0{\cdot}832$ |

$$\text{Inaccuracy (\%)} = \frac{\text{measured values} - \text{theoretical value}}{\text{theoretical value}} \times 100 \quad [8]$$

This process was repeated for nine days, and the inaccuracy was calculated taking the mean of the measured values. Good accuracy was observed for the sodium and the potassium ions, although the system tended to give values which were inferior to the theoretical ones. The results obtained for chloride ions were substantially below the theoretical ones.

The correlation between direct potentiometry and flame photometry and the indirect potentiometry (SMAC II) was also studied. One hundred serum samples of patients with concentrations varying from the studied components were assessed, in duplicate and by the three methods. The duplicates were distributed throughout the series, including lipaemic, icteric and haemolitic samples. In addition, 90 urine samples were processed on the same day, by both the Jokoo analyser and by flame photometry.

The correlation coefficient was calculated, the least squares line and the least squares regression according to Deming [9]. The results are shown in the table 4.

*Table 5. Detection limit calculation for a $\propto = 5\%$ risk.*

| $N = 30$ mmol/l | $\bar{x}$ | SD | Detection limit |
|---|---|---|---|
| Sodium | 9·16 | 1·66 | 12·48 |
| Potassium | 0·15 | 0·02 | 0·19 |
| Chloride | 12·7 | 1·03 | 14·76 |

*Detection limit*

The detection limit was taken to mean the least quantity, or concentration, of a substance that can be distinguished with a given probability of a reaction blank carried out under the same conditions [10]. This ideal reaction blank is executed by means of the Tris-orthophosphoric acid buffer at pH 7·35 and without the ions to be determined. The ionic concentrations of sodium, potassium and chloride were determined in a series of 10 determinations in 3 days (thus obtaining a total of 30 values per ion). For a risk $\propto$ to 5% and large samples ($N > 30$) the upper limit of the blank confidence interval was calculated as being $\bar{x} + 1{\cdot}97$, a value that corresponds to the detection limit for this risk. The results are shown in table 5.

*Linearity limits*

Linearity is expressed by the equation

$$y = a + bx \text{ [10]}.$$

Because it was impossible to obtain a standard containing the pure ion to be determined, sodium chloride and potassium chloride were used. On the basis of a stock standard prepared by weighing in Tris-orthophosphoric acid buffer at pH 7·35, a series in triplicate of decreasing dilutions was performed with the same buffer up to a total of 12 points, which were evaluated on three different days with a total of nine determinations per point. Then for each point the mean value and the standard deviation were calculated. The mean of the observed values corresponds to the ordinate axis, and the theoretical values to the abscissa axis. The bisector ($y = bx$, $b = 1$) is drawn and the linearity limits correspond to the maximum and minimum points of which the interval of confidence cut the bisector. The interval was calculated for a risk $\propto$ of 5% and 8 degrees of freedom by $X \pm 2{\cdot}306$ SD.

The least squares line and the correlation coefficient corresponding to the linearity interval was obtained. The results are shown in table 6.

*Estimation of the total error*

The errors corresponding to the inaccuracy and imprecision of a technique, which can sum up or compensate each other, can be calculated as follows:

$$\Delta - t \times \text{SD} \quad \text{and} \quad \Delta + t \times \text{SD} \qquad (10)$$

where $\Delta$ = inaccuracy, SD = standard deviation of the day-to-day imprecision, and $t$ = student $t$ value according to the risk $\propto$ and the degrees of freedom. Table 7 shows the results.

*Carry-over*

The evaluations of carry-over used three protocols [11–13] – each author reports on different aspects, all of which can affect the contamination between samples evaluated in series. For clarity, this report describes each of the three protocols, and results are given for each method.

Primary standards were prepared in the same way as those used in the linearity and inaccuracy study.

*Table 6. Linearity limits – calculated assuming* ∝ = *5% risk and 8° freedom. Linearity limits are indicated by asterisks. (N = 9.)*

| | | | | | | | | | | |
|---|---|---|---|---|---|---|---|---|---|---|
| **Sodium (mmol/l)** | | | | | | | | | | |
| Theoretical | 1000 | 800* | 500 | 250 | 125 | 62·5 | 31·2* | 15·6 | 7·8 | 3·9 |
| Observed | — | 809 | 494 | 241 | 123 | 64·4 | 34 | 24·3 | 16·1 | 12 |
| SD | — | 45·5 | 21 | 5·4 | 2·63 | 2·18 | 1·74 | 2·48 | 1·93 | 2·1 |
| Confidence limits | — | 704·2 | 445·7 | 228·6 | 117·1 | 59·4 | 30 | 18·6 | 11·6 | 7·15 |
| | | 914·2 | 542·5 | 253·4 | 129·3 | 69·4 | 38 | 30 | 20·6 | 16·8 |
| **Potassium (mmol/l)** | | | | | | | | | | |
| Theoretical | 64 | 40* | 20 | 10 | 5 | 2·5 | 1·25 | 0·65 | 0·31* | 0·15 |
| Observed | 50·34 | 39·26 | 19·3 | 9·46 | 4·84 | 2·37 | 1·26 | 0·68 | 0·38 | 0·28 |
| SD | 0·44 | 0·35 | 0·31 | 0·25 | 0·15 | 0·11 | 0·09 | 0·08 | 0·045 | 0·04 |
| Confidence limits | 49·3 | 38·4 | 15·58 | 8·88 | 4·49 | 2·12 | 1·05 | 0·5 | 0·28 | 0·19 |
| | 51·3 | 40·1 | 20·01 | 10·04 | 5·18 | 2·62 | 1·46 | 0·86 | 0·48 | 0·37 |
| **Chloride (mmol/l)** | | | | | | | | | | |
| Theoretical | 1080 | 864 | 540* | 270 | 135 | 67·5 | 33·75* | 16·87 | 8·45 | 4·21 |
| Observed | 866 | 698 | 509 | 258 | 127·8 | 65·4 | 35·4 | 23·5 | 17·8 | 4·6 |
| SD | 30·2 | 24·9 | 13·6 | 5·62 | 3·31 | 2·66 | 1·52 | 1·68 | 1·35 | 0·75 |
| Confidence limits | 796·4 | 640·4 | 478 | 245·2 | 120·2 | 59·3 | 31·9 | 19·6 | 14·7 | 12·9 |
| | 935 | 755·5 | 540·8 | 271·2 | 135·4 | 71·5 | 38·9 | 27·4 | 20·9 | 16·3 |

*Table 7. Total error estimation for a risk of* ∝ = *5% and 19° freedom.*

| $N = 20$ mmol/l | Theoretical | Observed | Δ | Imprecision (SD) | Total error variation |
|---|---|---|---|---|---|
| Sodium | 140 | 135·06 | −4·94 | 1·17 | −7·39 to −2·49 |
| Potassium | 4 | 3·84 | −0·16 | 0·021 | −0·2 to −0·116 |
| Chloride | 122·5 | 110·67 | −11·83 | 0·77 | −13·44 to −10·22 |

*Table 8. Carry-over studies according to Young–Gouchman's (interaction) and Bennet's protocols.*

| | Sequence $H \rightarrow L$ (mmol/l) | Inter-actions % | Carry-over % | Sequence $L \rightarrow H$ (mmol/l) | Carry-over % |
|---|---|---|---|---|---|
| Sodium | 196 49 | 0·55 | 0·82 | 49 141 | −0·14 |
| | 178 99 | 0 | 0 | 141 178 | −0·17 |
| | 159 81 | 0 | −0·11 | 99 159 | −0·37 |
| | 121 49 | 1·78 | 0·65 | 81 141 | −0·2 |
| | 196 141 | 0·2 | 0·20 | 49 196 | −0·39 |
| Potassium | 16 1 | 0·15 | 0 | 1 4 | −0·85 |
| | 8 2 | 0·21 | 0·51 | 4 16 | −1·69 |
| | 6 1·5 | 0·56 | 1·77 | 4 8 | 0·15 |
| | 16 4 | 0·38 | 1·6 | 1·5 6 | −1·1 |
| | 8 4 | 0 | 0·2 | 1 16 | −0·35 |
| Chloride | 190 60 | −0·39 | −0·66 | 135 190 | −0·58 |
| | 170 100 | 0 | −0·2 | 60 140 | −0·28 |
| | 160 80 | −0·15 | 0 | 140 170 | 0·89 |
| | 120 60 | 0·49 | −0·49 | 60 190 | −1 |
| | 190 140 | −1·77 | −0·29 | 80 140 | −0·42 |

*Interaction:* Interaction was evaluated according to Young and Gouchman [11]. A series of samples were analysed following the sequence below:

$$H_1 - H_2 - H_3 - L_1 - L_2 - L_3 - H_1 - H_2 - H_3$$

$H$ being the high sample concentration, and $L$ the low sample concentration. Each was evaluated in triplicate by applying the following formula:

$$\%\ \text{Interaction} = \frac{L_1 - L_3}{H_3 - L_3} \times 100$$

where $L_1$ is the contaminated sample, and $H_3$ is the contaminating agent.

*Carry-over*, according to Annette Bennet's protocol [12], uses the same sequence of samples as in the previous report. The following formula is applied:

$$\%\ \text{Carry-over} = \frac{L_1 - L_2}{L_2} \times 100$$

If the sample, $L$, is assumed to have been contaminated by the sample $H$.

$$\% \text{ Carry-over} = \frac{H_1 - H_2}{H_2} \times 100$$

if the sample $H$ is supposed to have been contaminated by the sample $L$.

Table 8 shows the results obtained for each of the concentration levels of the studied components, applying the two previous protocols.

For the majority of the studied concentration levels the contamination can be ignored because it was always less than 2%.

*Carry-over*, according to Broughton's protocol [13], uses the following sample sequence:

$$H_1 - H_2 - L_1 - L_2 - \ldots - L_1 - L_2$$

for 30 samples, $H$ being the high concentration level and $L$ the low. The following formula was used:

$$K = \frac{L_1 - L_2}{H_2 - L_2} \times 100$$

considering that, if $K$ is smaller than 2, there is no contamination.

The following results were obtained: $K$ = 0·038% for sodium; $K$ = 0% for potassium; $K$ = 1·47% for chloride. Contamination, then, is practically negligible for the three studied components.

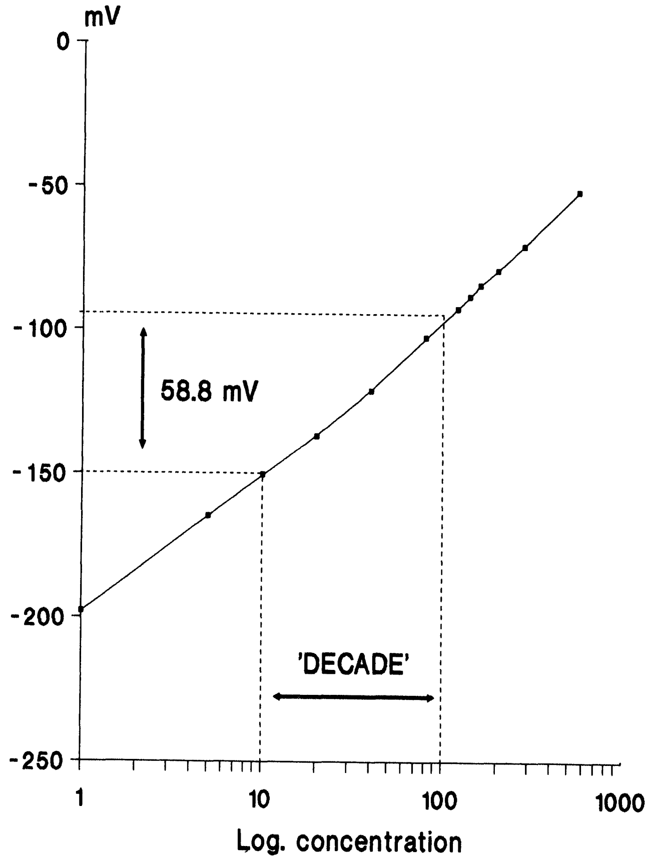

*Figure 1. Sodium electrode slope study.*

*Drift*

A commercial control was used – the level of which coincided with the physiological values for each component. Controls were evaluated at the beginning of a series of samples (after the first calibration), at the end of this series of samples (prior to the recovery of the base-line and to the second calibration) and, finally, at the end of the day's work after having processed various sample series and having realized various calibrations. This was performed over six days.

Therefore, the drift has been studied both in a series and also during the day's work. The values obtained at the beginning of the series were compared to those at the end of the series. In addition, those values obtained at the beginning of the series were compared to those from the end of the day's work.

Table 9 shows the results obtained from both studies – the drift was negligible.

*Table 9. Drift study. ($N$ = 6.)*

| | Calibration drift | | |
|---|---|---|---|
| | Initial mmol/l | Final mmol/l | Drift % |
| Sodium | 138·2 | 138·2 | 0 |
| Potassium | 3·63 | 3·61 | −0·55 |
| Chloride | 110·97 | 112·28 | 1·18 |

| | Day's work drift | | |
|---|---|---|---|
| | Initial mmol/l | Final mmol/l | Drift % |
| Sodium | 140·1 | 140·4 | 0·21 |
| Potassium | 3·095 | 3·077 | −0·58 |
| Chloride | 118·1 | 119·15 | 0·89 |

*Electrode slope study*

For the electrode slope study, the same stock standards and solutions with decreasing concentrations in the linearity study were used. The negative pole of the voltimeter was connected to the external reference electrode, and the positive pole to the electrode under evaluation. The results were the concentrations and the corresponding responses in mV from the voltimeter (see figures 1–3). The abscissa axis is the concentration logarithm and the ordinate axis the corresponding mV-value; the slope of every electrode was obtained by giving it the mV-values corresponding to 10-times increase in the concentration of the ion (a 'decade') [14]. Considering the theoretical value of the slope of 59 mV as ideal for monovalent ions, acceptable results are observed for the three electrodes.

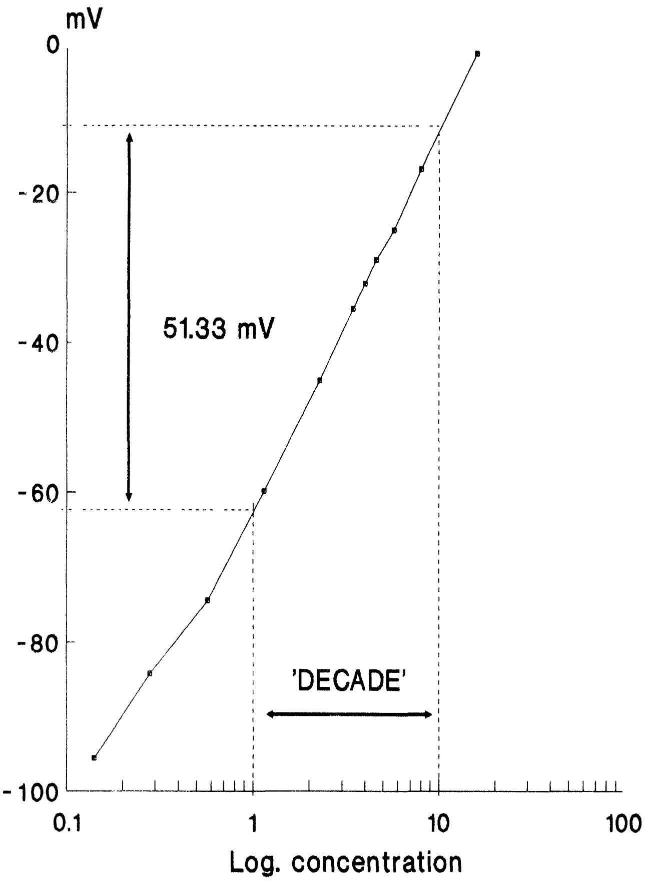

Figure 2. Potassium electrode slope study.

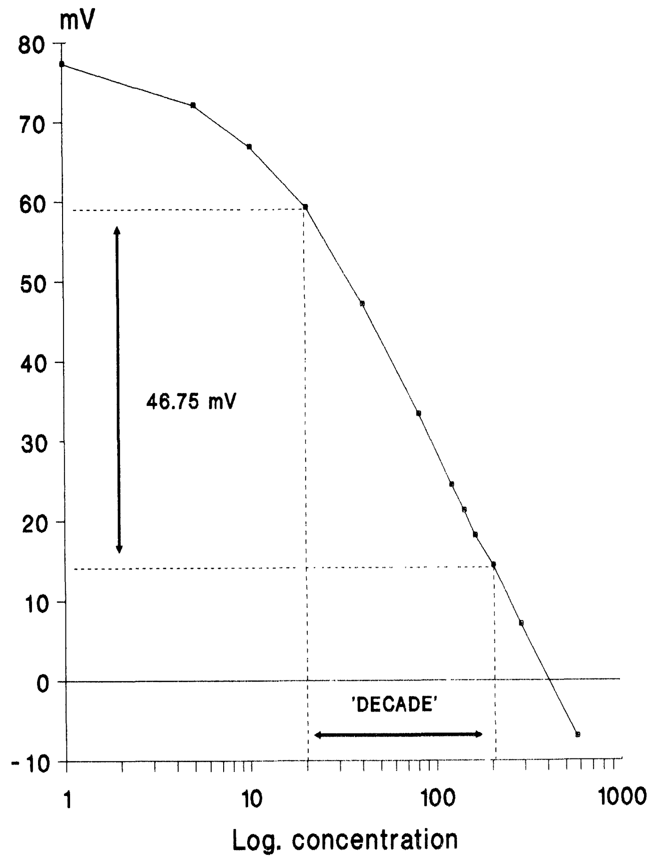

Figure 3. Chloride electrode slope study.

*Electrode response study*

The response of each electrode was studied separately, using a serum pool and concentrating on the response time and the stability of the steady state obtained [15]. For that purpose an analogue registrator was connected to the outlet of every electrode. The stability of the steady state was good for all three electrodes, with a very short response time – less than 1 s – in achieving the plateaus and with a total duration of less than 22 s.

The results are shown in figures 4, 5 and 6.

*Sample temperature effect*

Three commercial controls were evaluated on being removed from the refrigerator (≃4 °C), and after being stored at room temperature (≃26 °C). The controls were all evaluated after 5, 15 and 30 min, and always within the same analytic series.

The percentage of variation for each concentration level was calculated, with reference to the concentration which was evaluated initially (≃4 °C). Table 10 shows the percentages obtained in every measurement with reference to the initial values.

*Effect of pH on electrode response*

Three stock standards of identical ionic concentration were prepared. The standards all had different pHs: 8·48, 5·17 and 7·37 with Tris-orthophosphoric acid buffer. A series of decreasing dilutions was then executed, with a constant pH being maintained over the series. The concentrations were evaluated in duplicate; this value was used on the ordinate axis and the corresponding theoretical value on the abscissa axis. By joining the intersection points of each series with identical pHs, the deviations suffered by the ion values as a consequence of pH variation was plotted – see figures 7, 8 and 9.

In a similar way the pH levels of interest to clinical practice were measured, and a minimum influence within the pH-margins were observed – see table 11.

*Interference studies*

*Influence of the sodium ion on the potassium ion electrode.* A series of solutions were prepared with a constant concentration of 4 mmol/l of potassium and decreasing sodium concentrations of 200, 100, 50 and 8 mmol/l, in Tris-orthophosphoric acid buffer with 1 ml/l of a surfactant. Each solution was evaluated five times, the mean value taken and the effect of the sodium ion concentration over the constant value of the potassium ion calculated. The concentrations of this series were also evaluated by flame photometry, in order to check the accuracy of the concentration of each ion.

In figure 10 the concentrations of the sodium ion are shown on the abscissa and those of the potassium ion on the ordinate – thus the evolution of the potassium ion

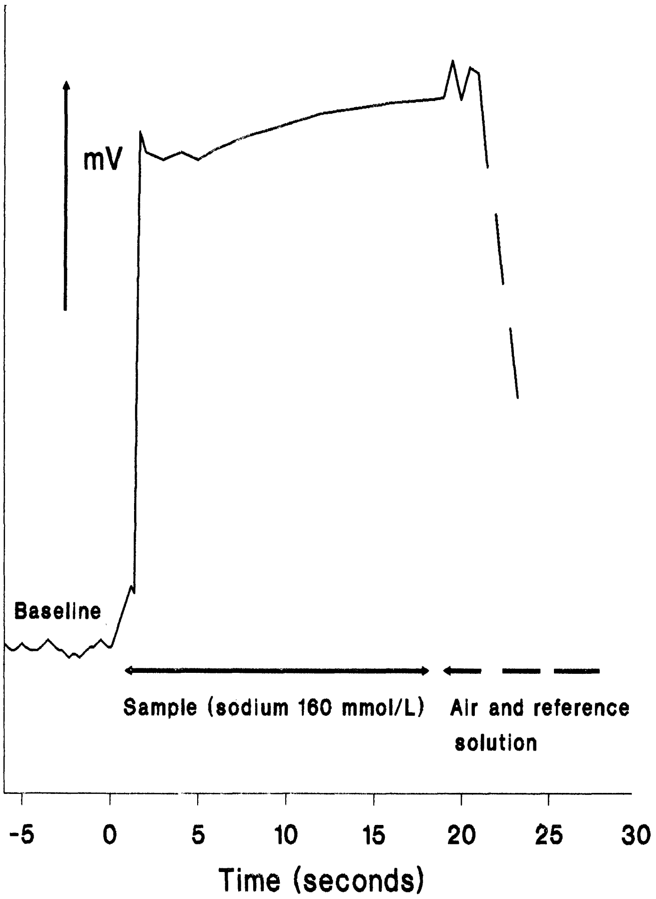

*Figure 4. Sodium electrode response study.*

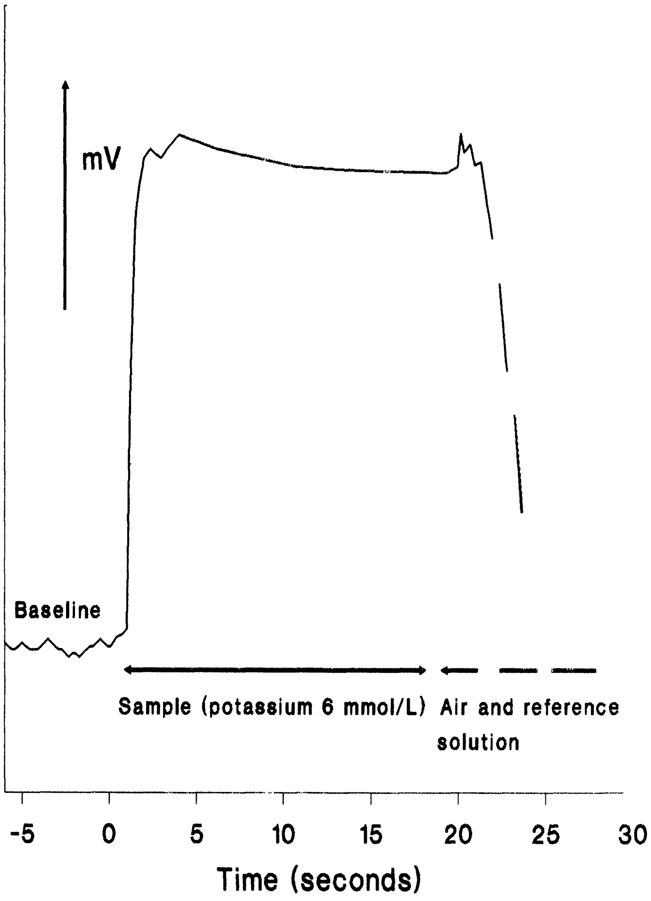

*Figure 5. Potassium electrode response study.*

values against the variations of the sodium ion concentration, can be observed. Between the margins corresponding to 50 mmol/l and 200 mmol/l of sodium, the effect on the potassium ion has no significance.

The same sample series was processed by the ion analyser, following the protocol for urine samples, and no effect was found on the series.

*Effect of the potassium ion on the sodium ion electrode.* For a constant concentration of 125 mmol/l sodium, potassium concentrations of 250, 125, 62, 30, 15, 7·5 and 3·2 mmol/l in Tris-orthophosphoric acid buffer were prepared. Each solution was evaluated five times, and the mean value was determined later.

The potassium ion concentration effect on a constant sodium ion concentration was also calculated. The accuracy of the concentrations of both ions in this series was checked by flame photometry. Figure 11 shows the potassium ion values on its abscissa and the sodium ion values, found as a result of the different potassium ion concentrations, on its ordinate axis. The influence of the potassium ion on the electrode of the sodium ion is insignificant.

*Influence of the lithium ion as a component of the anticoagulant agent.* The human serum pool was used for serial dilutions of the samples in tubes with lithium heparin. The lithium heparin dilutor effect can be considered insignificant, since the volume used is very small. Thus a lithium concentration gradient was obtained. Seven dilutions were obtained and each was evaluated for concentration of sodium ions, potassium ions, chloride ions and lithium.

Figure 12 gives the concentration percentage variations of the ions on its ordinate axis, and the lithium ion concentration on its abscissa. A reduction in potassium ion concentration can be observed with an increase in lithium ions.

*Influence of the ammonium ion.* A human serum pool was prepared. Ammonium chloride was dissolved to give a final concentration of 100 mmol/l, and by means of another pool fraction, eight solutions of decreasing concentration of ammonium chloride were prepared. The ammonium ion concentration in each solution was evaluated in line with the previous method. At the same time, the sodium and the potassium ion concentrations were evaluated in the original pool and in the ammonium ion solutions.

Figure 13 gives the sodium and potassium concentrations on its ordinate axis, against ammonium ion concentrations. Neither ion is subject to any interference from ammonium ions at physiopathological concentrations. Significant interference was seen with experimental concentrations of ammonium higher than 10 mmol/l (1·38%) and 100 mmol/l (28·7%), which might affect the

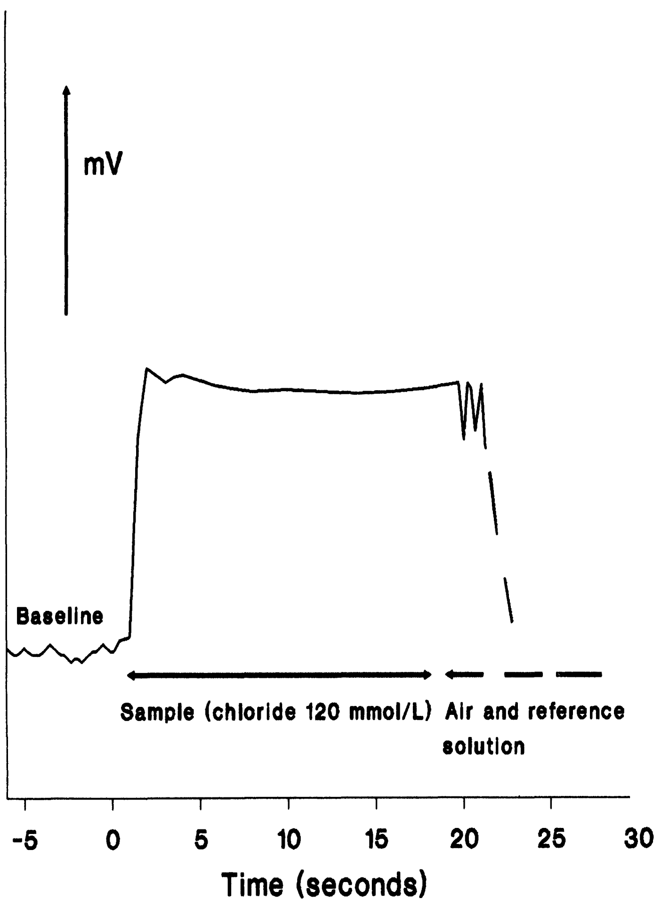

*Figure 6. Chloride electrode response study.*

potassium evaluations for urine samples with high ammonium ion concentrations.

*Effect of halogens on the chloride electrode.* For a constant concentration of 125 mmol/l chloride, decreasing bromide concentrations of 250, 125, 62, 30, 15, 7·5 and 3·2 mmol/l in Tris-orthophosphoric acid buffer were prepared. Each solution was evaluated five times and the mean and the bromide ion concentration impact on a constant chloride concentration were calculated.

Bromide values are shown on the abscissa axis of figure 14, and the ordinate axis gives the chloride values found as a result of the different bromide concentrations. There is a geometrically proportional influence of bromide on chloride, which, in spite of the fact it is small at lower concentrations, should be taken into account for patients who are receiving bromide and other halogens in substantial amounts.

*Influence of lipid and protein concentration.* The protein concentration effect of direct potentiometry, indirect potentiometry and flame photometry on sodium and potassium ions has also been studied.

A human albumin solution at 20% was used which was poor in ions. A lipaemic solution which was 10% free of ions was also used. Decreasing dilutions were prepared, in duplicate, maintaining a fixed ion concentration and processing each of them following three survey methods.

Tables 12 and 13 show the variation of direct potentiometry in relation to indirect potentiometry and flame photometry.

Due to the effect of the sample content of plasmatic water [16], some sodium and potassium ion values could be observed to be inversely proportional to the protein concentration in those methods that employ dilution relating to direct potentiometry. The same effect, but to a minor degree, could be seen in the 10% lipid series (long-chain triglyceride).

*Whole blood samples*

*Haematocrit influence.* The eventual haematocrit influence on the sodium and potassium and chloride ion concentration evaluation were checked. Starting from blood samples obtained with lithium heparin anticoagulant of the same patient, a series of whole blood samples was prepared with a haematocrit varying from 4 to 70%. This preparation was made by adding or subtracting plasma, after the corresponding phases of centrifugation, until the desired haematocrits were achieved.

In this assay, very small differences in the chloride ion, potassium ion and sodium ion evaluations were observed, except for the samples with a haematocrit higher than 65%, where the potassium ion concentration value was subject to a strong increase. This effect could result from the way the sample flows through the capillary which

*Table 10. Study of the influence of temperature on concentration (mmol/l).*

| | Taken out of fridge (4 °C) | Time (min) at room temperature (26 °C) | | | | | |
|---|---|---|---|---|---|---|---|
| | | 5 | % Δ | 15 | % Δ | 30 | % Δ |
| Sodium | 121·3 | 121·3 | 0 | 121·9 | 0·49 | 122·2 | 0·74 |
| | 138·9 | 139·4 | 0·36 | 140 | 0·79 | 141·3 | 1·73 |
| | 153·1 | 154·1 | 0·65 | 155·3 | 1·44 | 155·8 | 1·76 |
| Potassium | 3·18 | 3·16 | −0·63 | 3·15 | −0·94 | 3·14 | −1·26 |
| | 6·26 | 6·24 | −0·32 | 6·24 | −0·32 | 6·25 | −0·16 |
| | 7·03 | 7·03 | 0 | 7·05 | 0·28 | 7·06 | 0·43 |
| Chloride | 96·8 | 97·1 | 0·31 | 97·2 | 0·41 | 97·1 | 0·31 |
| | 117·3 | 117·1 | −0·17 | 117·1 | −0·17 | 117·1 | −0·17 |
| | 124·9 | 125·2 | 0·24 | 125·5 | 0·48 | 124·6 | 0·24 |

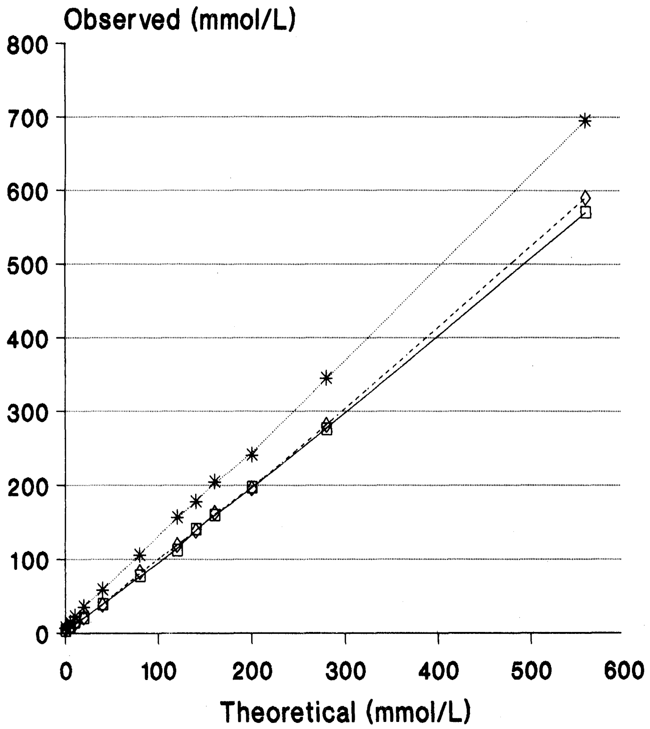

*Figure 7. Sample pH influence on sodium electrode. □, pH 8·48; ◇, pH 7·37; ✳, pH 5·17.*

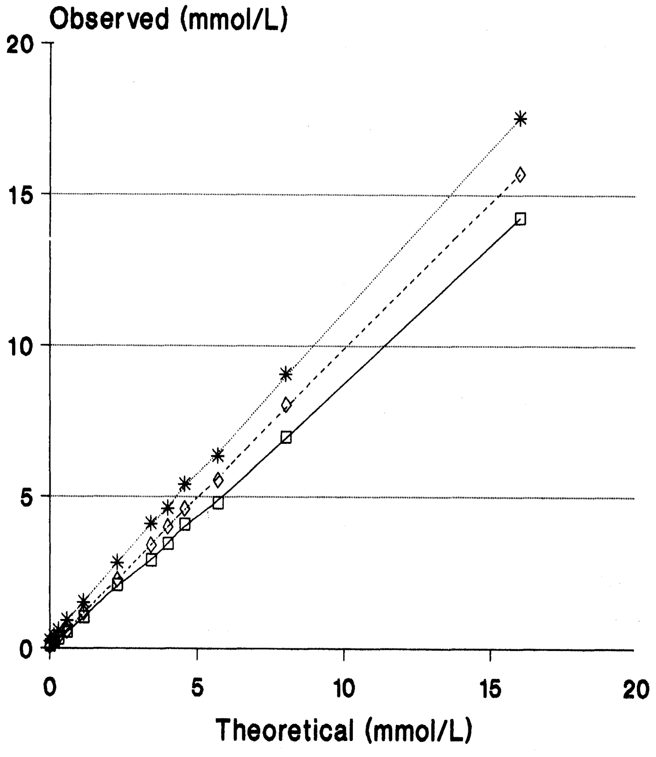

*Figure 8. Sample pH influence on potassium electrode. □, pH 8·48; ◇, pH 7·37; ✳ pH 5·17.*

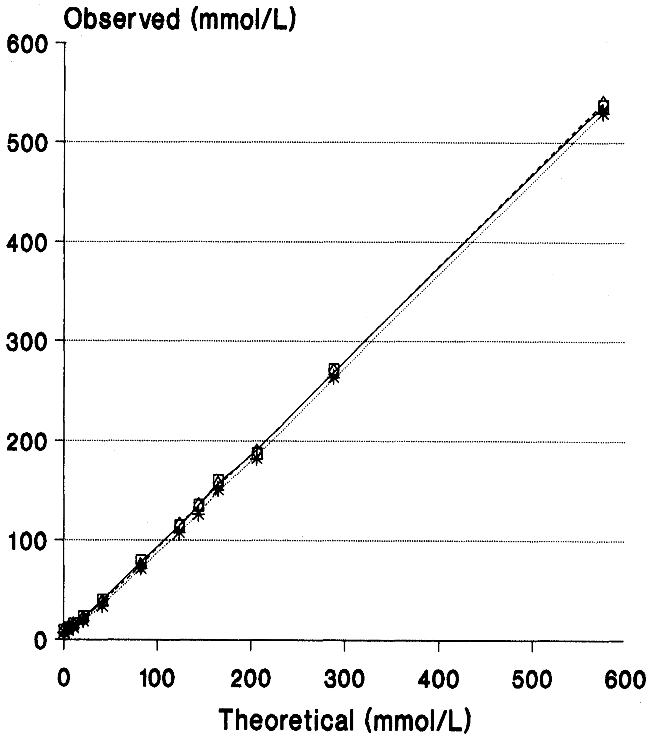

*Figure 9. Sample pH influence on chloride electrode. □, pH 8·48; ◇, pH 7·37; ✳ pH 5·17.*

connects with the measuring chamber, where the haematites, owing to their number, could suffer lysis. Thus, releasing the intraeritrocitary potassium ion would increase the total potassium ion concentration (see table 14).

### Conclusions

The evaluation described here includes guidelines for ion-selective electrode analyser evaluations. The following results were found from the assays reported:

*Table 11. Dependence of ionic concentration (mmol/l) variation on physiopathological pH values.*

| | Theoretical | Observed values | | | Δ pH 6.9–7·4 |
|---|---|---|---|---|---|
| | | pH 6·9 | pH 7·4 | pH 7·8 | |
| Sodium | 120 | 122·6 | 118·8 | 116·9 | 5·7 |
| | 140 | 140·3 | 136·6 | 134·6 | 5·7 |
| | 160 | 159·9 | 156 | 153·3 | 6·6 |
| Potassium | 2·5 | 2·45 | 2·45 | 2·44 | 0·01 |
| | 4 | 3·85 | 3·85 | 3·83 | 0·02 |
| | 6 | 5·86 | 5·85 | 5·81 | 0·05 |
| Chloride | 122·5 | 109·2 | 109·8 | 110·3 | 1·1 |
| | 144 | 130·4 | 131 | 131·7 | 1·3 |
| | 166 | 154·2 | 155·4 | 155·7 | 1·5 |

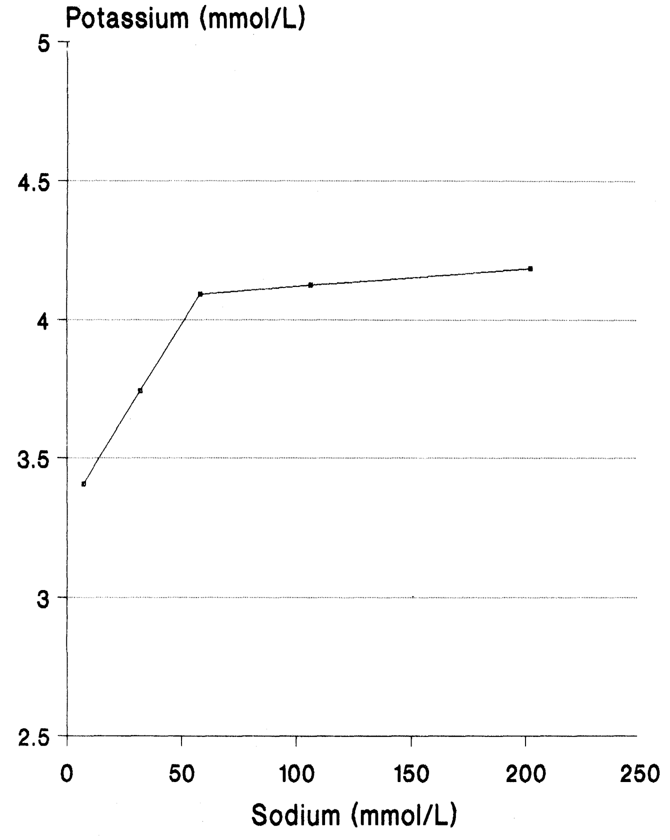

*Figure 10. Influence of the sodium ion on the potassium electrode.*

(1) The within-run imprecision for the Jokoo-ION 150 AC was smaller than 1%, and the day-to-day imprecision less than 2%.

(2) The inaccuracy relating to the primary standard values was good for the sodium and potassium ion, and acceptable for the chloride ion.

(3) The detection limit for the sodium ion is 12·48 mmol/l, for the potassium ion 0·19 mmol/l and for the chloride ion 14·76 mmol/l.

(4) The linearity limits are higher than 500 mmol/l for the sodium ion and chloride ion, and more than 45 mmol/l for the potassium ion.

(5) Carry-over and drift are negligible (smaller than 2%). The results were not significantly affected by sample temperature.

(6) In the study of the electrode slope, some values close to 59 mV were obtained, which is considered to be the optimal value for monovalent ions. The response time is also very short (less than 1 s), and stability is maintained for 22 s.

(7) Significant variations due to pH were observed on both ions (sodium and potassium).

(8) The interference study showed a slightly negative interference on the potassium ion due to the increment of the lithium ion. Only high ammonium ion concentrations cause a positive interference on the potassium ion. It was also observed that there

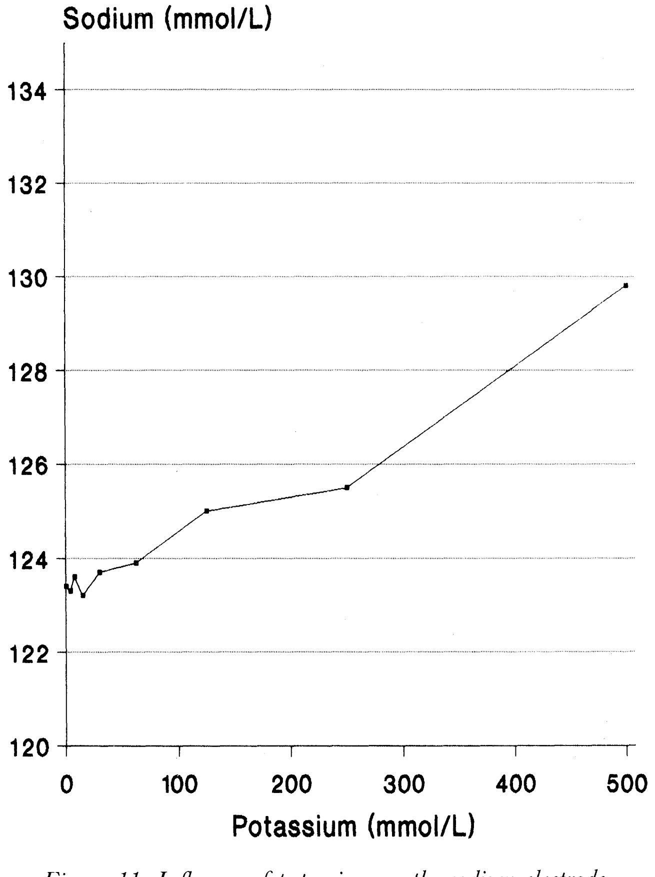

*Figure 11. Influence of potassium on the sodium electrode.*

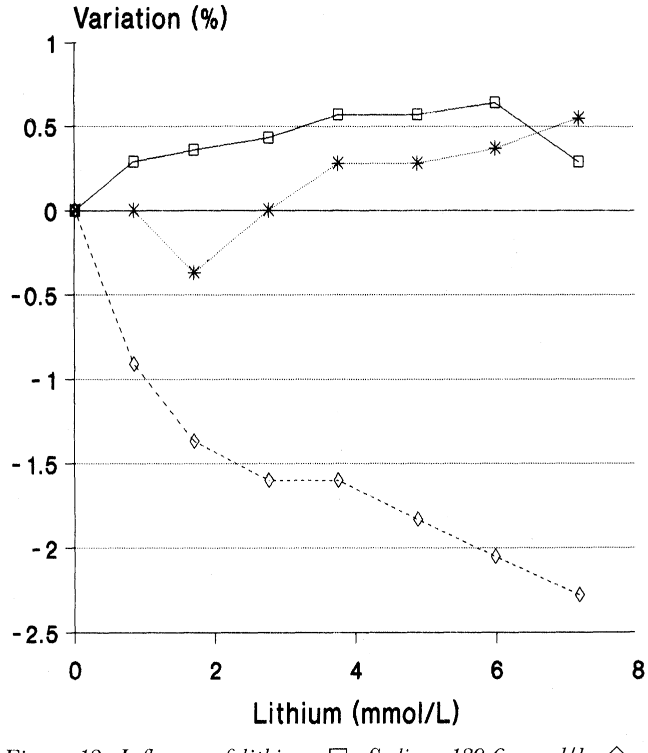

*Figure 12. Influence of lithium.* □, *Sodium 139·6 mmol/l;* ◇, *potassium 4·4 mmol/l;* ✳ *chloride 109 mmol/l.*

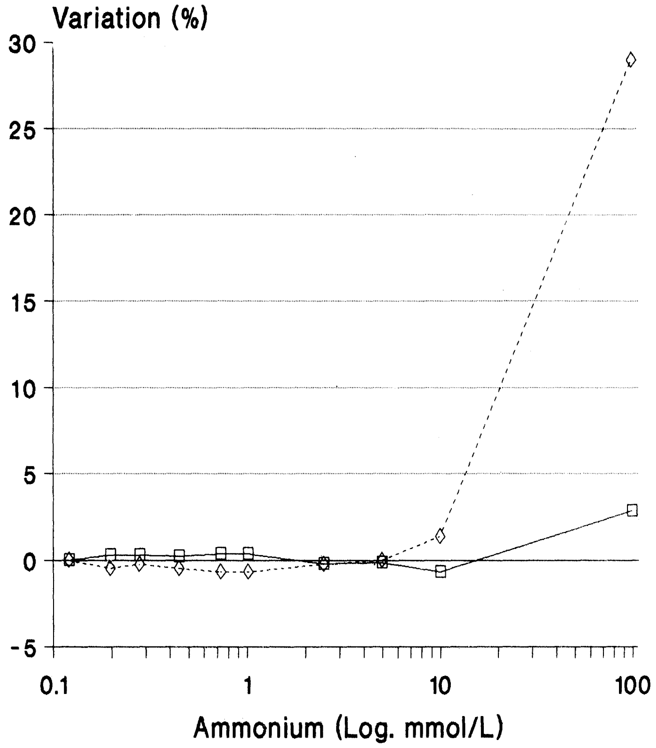

*Figure 13. Influence of ammonium on the sodium and potassium electrodes.* □, *Sodium 135·9 mmol/l;* ◇, *potassium 4·3 mmol/l.*

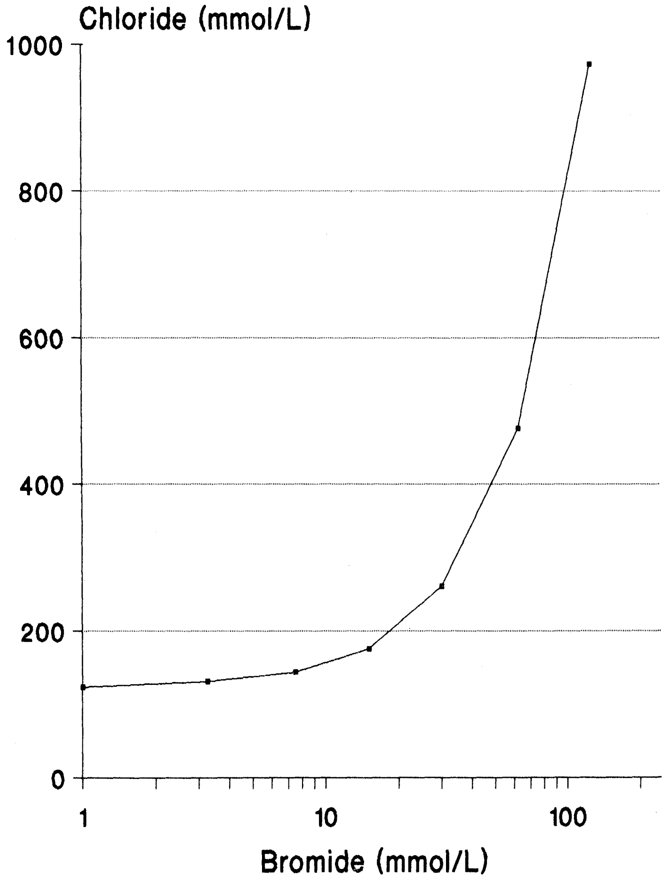

*Figure 14. Influence of bromide on the chloride electrode.*

*Table 12. Influence of lipid concentration. Differences are given in sodium concentration between direct potentiometry and flame photometry and indirect potentiometry for solutions which are free of lipids.*

| Tri-glycerides mmol/l | Direct potentio-metry | Flame photo-metry | Δ mmol/l | Indirect potentio-metry | Δ mmol/l |
|---|---|---|---|---|---|
| 0 | 140·7 | 143·6 | — | 141 | — |
| 0·3 | 140·6 | 143 | 0·5 | 140 | 0·9 |
| 0·6 | 140·3 | 142·4 | 0·8 | 141 | −0·4 |
| 1·75 | 140 | 142 | 0·9 | 140 | 0·3 |
| 2·5 | 140·9 | 142·3 | 1·5 | 140 | 1·2 |
| 5 | 141·8 | 142·4 | 2·3 | 140 | 2·1 |
| 7·5 | 144·9 | 142·7 | 5·1 | 141 | 4·2 |
| 10 | 143·1 | 140·8 | 5·2 | 140 | 4·4 |

*Table 13. Influence of protein. Differences are given in sodium concentration between direct potentiometry/flame photometry and indirect potentiometry for solutions free of proteins. The sodium concentration is not fixed, as the albumin concentrate* has sodium in it.*

| Protein g/l | Direct potentio-metry | Flame photo-metry | Δ mmol/l | Indirect potentio-metry | Δ mmol/l |
|---|---|---|---|---|---|
| 0 | 134·9 | 139·6 | — | 135 | — |
| 25·6 | 139·2 | 142·8 | 1·1 | 136 | 3·3 |
| 46·9 | 144·2 | 146·7 | 2·2 | 142 | 2·3 |
| 65·8 | 147·6 | 149·9 | 2·4 | 142 | 5·7 |
| 79·8 | 154 | 155·2 | 3·5 | 146 | 10·1 |
| 99·3 | 160·4 | 160 | 5·1 | 147 | 13·5 |
| 127·7 | 167·9 | 163·5 | 9·1 | 156 | 12 |
| 263* | 57·1 | 48·6 | — | 46 | — |

*Table 14. Influence of haematocrits.*

| Haemato-crit | Sodium (mmol/l) | Sodium Δ % | Potassium (mmol/l) | Potassium Δ % | Chloride (mmol/l) | Chloride Δ % |
|---|---|---|---|---|---|---|
| Plasma | 141·4 | — | 4·05 | — | 108 | — |
| 5% | 141·7 | 0·21 | 4·09 | 0·99 | 108·6 | 0·55 |
| 18% | 141 | −0·28 | 4·07 | 0·49 | 107·5 | −0·46 |
| 35% | 143·6 | 1·5 | 4·03 | −0·49 | 107 | −0·93 |
| 45% | 142·8 | 0·99 | 4·09 | 0·99 | 106·2 | −1·66 |
| 60% | 140·9 | −0·35 | 4·11 | 1·48 | 105·2 | −2·59 |
| 70% | 143 | 1·13 | 4·42 | 9·14 | 107·6 | −0·37 |

was a slight positive interference of the bromide ion on the chloride ion.

(9) The effect of high protein or lipid concentrations produced ionic concentrations proportionally more elevated in direct potentiometry, than in indirect potentiometry and in flame photometry.

(10) In the evaluation of whole blood samples significant increases of the potassium values were observed, starting from a haematocrit of 60% on.

### References

1. Tietz, N. W., *Química Clínica Moderna* (Interamericana, S. A., Mexico, 1972).
2. Maron, S. H. and Prutton, C. F., *Fundamentos de Fisicoquímica* (Limisa, S. A., Mexico, 1972).
3. Oesch, V., Ammann, D. and Simon, W., *Clinical Chemistry,* **32** (1986), 1448.
4. Rao, J., Pelavin, H. M. and Morgenstern, S., *Advances in Automated Analysis: Technicon International Congress 1972. Vol. 1: Clinical Chemistry Methods* (Mediad, Inc., New York, 1973), p. 33.
5. Lavinia, A., Pioda, R., Simon, W., Bosshard, H. R. and Curtius, H. C., *Clinica Chimica Acta,* **39** (1970), 289.
6. Morgernstern, S., Rush, R. and Lehman, D., *Advances in Automated Analysis. 1972. Technicon International Congress Vol. 1* (Mediad, Inc., White Plains, New York, 1973).
7. *Manual de Instrucciones Flame photometer 943* (Instrumentation Laboratory S.p.A., Vialle dell'Industria, 3 Padermo, Italy).
8. Société Française de Biologie Clinique. Commission 'Validation de techniques'. *Dictionaire des termes à l'usage de la validation de techniques. Annales de biologie clinique,* **44** (1986), 679.
9. Payne, R. B., *Clinical Chemistry,* **30** (1984), 807 (letter).
10. Société Française de Biologie Clinique. Commission 'Validation de techniques'. *Protocole de validation de techniques. Annales de biologie clinique,* **44** (1986), 686.
11. Young, D. S. and Gouchman, N., Methods for assuring quality data from continuous flow analyzers. *Standard Methods of Clinical Chemistry,* **7** (Academic Press, New York, 1972), 293.
12. Bennet, A., Gartelmann, D., Ason, J. I. and Owen, J. A., *Clinica Chimica Acta,* **29** (1970), 161.
13. Broughton, P., *Journal of Automatic Chemistry,* **6** (1984), 94.
14. Sachs, Ch., *Annales de biologie clinique,* **43** (1985), 715.
15. Sachs, Ch., Données pratiques pour l'éssai automates 'electrometriques'. *I.S.B.,* **5** (1982), 397.
16. Sachs, Ch. and Truchaud, A., Potentiometrie 'Directe' contre Potenyiometric 'Indirecte' a propos d'une guerre picrocholine en electrometrie. *I.S.B.,* **9** (1983), 103.

